# BFLIDS: Blockchain-Driven Federated Learning for Intrusion Detection in IoMT Networks

**DOI:** 10.3390/s24144591

**Published:** 2024-07-15

**Authors:** Khadija Begum, Md Ariful Islam Mozumder, Moon-Il Joo, Hee-Cheol Kim

**Affiliations:** Institute of Digital Anti-Aging Healthcare, Inje University, Gimhae 50834, Republic of Korea; khadijahappy.cse@gmail.com (K.B.); arifulislamro@gmail.com (M.A.I.M.); joomi@inje.ac.kr (M.-I.J.)

**Keywords:** blockchain, Internet of Medical Things (IoMT), intrusion detection system, federated learning, security, privacy

## Abstract

The Internet of Medical Things (IoMT) has significantly advanced healthcare, but it has also brought about critical security challenges. Traditional security solutions struggle to keep pace with the dynamic and interconnected nature of IoMT systems. Machine learning (ML)-based Intrusion Detection Systems (IDS) have been increasingly adopted to counter cyberattacks, but centralized ML approaches pose privacy risks due to the single points of failure (SPoFs). Federated Learning (FL) emerges as a promising solution, enabling model updates directly on end devices without sharing private data with a central server. This study introduces the BFLIDS, a Blockchain-empowered Federated Learning-based IDS designed to enhance security and intrusion detection in IoMT networks. Our approach leverages blockchain to secure transaction records, FL to maintain data privacy by training models locally, IPFS for decentralized storage, and MongoDB for efficient data management. Ethereum smart contracts (SCs) oversee and secure all interactions and transactions within the system. We modified the FedAvg algorithm with the Kullback–Leibler divergence estimation and adaptive weight calculation to boost model accuracy and robustness against adversarial attacks. For classification, we implemented an Adaptive Max Pooling-based Convolutional Neural Network (CNN) and a modified Bidirectional Long Short-Term Memory (BiLSTM) with attention and residual connections on Edge-IIoTSet and TON-IoT datasets. We achieved accuracies of 97.43% (for CNNs and Edge-IIoTSet), 96.02% (for BiLSTM and Edge-IIoTSet), 98.21% (for CNNs and TON-IoT), and 97.42% (for BiLSTM and TON-IoT) in FL scenarios, which are competitive with centralized methods. The proposed BFLIDS effectively detects intrusions, enhancing the security and privacy of IoMT networks.

## 1. Introduction

The widespread adoption of Internet of Things (IoT) devices has ushered in a new era of connectivity, revolutionizing sectors such as healthcare, transportation, and manufacturing. In the healthcare industry, where maintaining data confidentiality and integrity is essential, any breach or tampering can have severe repercussions for patients [1]. For example, unnoticed changes to medical records could lead to severe health risks or even accidental deaths [2]. The increasing use of Internet of Medical Things (IoMT) devices has also exposed significant cybersecurity issues [3]. Traditional security measures often fall short because many IoT devices have limited computing power, memory, and battery life. Additionally, conventional IoMT systems face challenges like single points of failure and vulnerabilities to unwanted intrusions.

**Figure 1:**
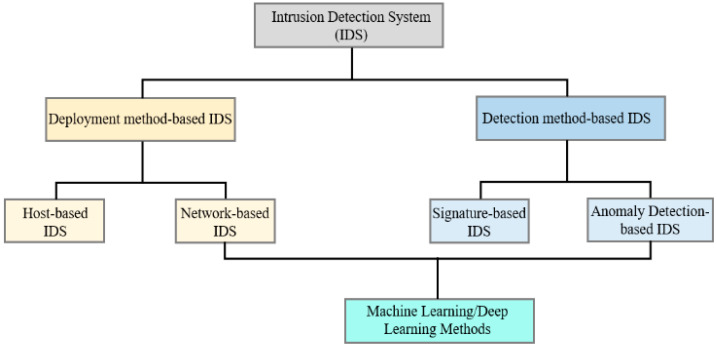
Deployment and detection-based Intrusion Detection Systems.

An Intrusion Detection System (IDS) works as a security system for networks, designed to detect unusual activities and alert administrators. It examines the metadata in network packets and decides whether to allow or block traffic based on predefined rules [4]. IDSs provide three crucial security measures: (i) data confidentiality: ensuring data are stored securely; (ii) data availability: making sure data are accessible to authorized users; and (iii) data integrity: checking that data are accurate and consistent. Intrusion detection techniques are initially divided into two main categories: deployment-based and detection-based. Deployment-based IDSs can be either host-based, monitoring individual devices, or network-based, monitoring network traffic. Detection-based IDSs can be signature-based, identifying known threats, or anomaly-based, detecting unusual patterns [5,6]. This results in four total IDS methods, combining deployment and detection techniques, as illustrated in Figure 1.

ML approaches have immensely improved the ability of IDSs to identify potential threats by using various algorithms, such as neural networks, to analyze network traffic [5,7,8,9]. Traditionally, industries would manage and analyze data from IoMT devices by storing it in a central database. While this centralized approach simplifies management, it raises privacy and security concerns. Recent studies have highlighted vulnerabilities in ML models, where subtle and untraceable changes to input data can lead to incorrect predictions, deceiving the ML algorithms. Additionally, conventional centralized methods often struggle in scenarios where private data are not readily available. Furthermore, these systems require the aggregation of sensitive data from IoMT devices in a central server for analysis, which poses significant privacy risks. This is especially concerning in healthcare settings where patient data confidentiality is paramount. Furthermore, as the number of IoMT devices increases, centralized systems struggle to scale efficiently. The centralized processing unit becomes a bottleneck, resulting in increased latency and reduced detection accuracy.

Due to the volume and sensitivity of information shared by IoMT devices, the aforementioned issues can become even more severe. Federated Learning (FL) offers a solution by providing a decentralized learning approach that addresses the privacy concerns of traditional centralized ML methods. Introduced in 2016, FL allows end devices (clients) to share only the updates of a global model, which are then aggregated by a central unit (aggregator) [10]. This method enhances user privacy by never sharing the actual data. FL enables machine learning to derive insights from diverse datasets from different locations, allowing multiple parties to collaborate on model development without exposing sensitive information. This approach exposes the shared models to a broader range of data compared to a single central entity, improving the robustness and accuracy of the models [11]. However, existing FL-based IDS solutions also face several challenges. First, IoMT devices generate diverse and heterogeneous data, making it difficult to develop a universal model that performs well across different devices and scenarios. Second, FL systems are susceptible to adversarial attacks, where malicious devices can manipulate model updates to degrade the overall model performance. Lastly, IoMT devices often have limited computational and storage resources, which restricts their ability to participate effectively in Federated Learning.

Several studies have attempted to address these challenges, but gaps remain. While FL enhances privacy, it does not inherently ensure the integrity and immutability of the model updates. However, few studies have explored this integration in the context of IDSs for the IoMT. Additionally, most existing studies utilize basic ML algorithms for intrusion detection, overlooking the potential of advanced techniques that can capture complex relationships and temporal patterns in IoMT data, thus leading to more accurate and robust intrusion detection. Furthermore, current FL-based IDS solutions often do not address the scalability and efficiency required for large-scale IoMT deployments, making it crucial to optimize the communication and computation overhead in FL systems for practical implementation.

Blockchain offers several advantages for distributed data storage and safeguarding IoMT networks [12]. As a peer-to-peer distributed ledger, blockchain records transactions sequentially and in a tamper-proof manner, keeping users at the center of the ecosystem [13]. Encrypted nodes manage and store blocks of time-stamped immutable data records linked by cryptographic hashes. This setup is crucial for defending IoMT systems from various attacks as the current environment relies heavily on precise ML techniques [14]. Smart contracts (SCs) are self-executing contracts with the terms of the agreement directly written into code. They autonomously perform contract-related tasks and provide evidence of fulfillment [15]. SCs are essential for managing all interactions and activities in a blockchain network. However, storing large volumes of data on a typical blockchain is impractical and expensive. The InterPlanetary File System (IPFS) provides an effective solution by enabling the storage of substantial amounts of data like blockchain [16]. Additionally, MongoDB offers a decentralized infrastructure where authorized nodes can develop and manage distributed applications [17]. The combined use of IPFSs and MongoDB as a decentralized storage system is highly beneficial for an IDS solution in the IoMT, ensuring efficient and secure data management. Because of all these advantages, blockchain can complement FL by providing a secure and transparent ledger for recording model updates and transactions.

While blockchain has been proposed as a solution to enhance the security and integrity of FL systems, studies by Yu et al. [18] and Bobde et al. [19] have highlighted significant challenges, including integration overheads and scalability issues. Most studies typically use energy-intensive consensus mechanisms, which are not suitable for resource-constrained IoMT devices. Moreover, the existing research often overlooks the need for data confidentiality and the conflict between blockchain transparency and privacy requirements, which are crucial for maintaining the integrity of sensitive medical data in IoMT networks. Finally, the interoperability between different blockchain platforms and IoMT devices remains a significant challenge that has not been adequately addressed. Current solutions lack the necessary cross-chain technologies and standardization efforts to ensure a seamless interaction across diverse systems [20].

Our research addresses these critical gaps by proposing a blockchain-enabled Federated Learning approach that integrates advanced ML for IDS solutions. We develop efficient blockchain integration methods to ensure the security and privacy of IoMT data. Our approach decentralizes the training of IDS models by conducting learning and inference on local clients, ensuring that sensitive information is never shared externally. Instead, only the model parameters are shared with a central authority responsible for aggregating and enhancing the global model in subsequent Federated Learning iterations.

The contributions that this work achieved are listed below:

- Novel blockchain-based Federated Learning approach: We propose a secure Federated Learning method for IoMT intrusion detection, which trains models on local clients and federates their learning.
- Performance evaluation: We compare our Federated Learning method to traditional centralized ML methods using various popular datasets that cover numerous real-world attack scenarios.
- Experimental outcomes: Our results show that distributing and aggregating models in a Federated Learning environment is competitively effective compared to centralized methods. This analysis and the proposed strategy offer valuable insights for future research in this area.

The remaining sections of this article are structured as follows. In Section 2, we provide a literature review of the latest advancements in Federated Learning (FL) related to the IoT and intrusion detection. Section 3 details the proposed method and its components. Section 4 presents and thoroughly discusses the experimental results and evaluation. Finally, Section 5 concludes this article by highlighting several potential future research directions.

## 2. Related Works

Every day, new types of attacks are developed by malicious intruders. To counter these threats, Intrusion Detection Systems (IDSs) must be capable of quickly identifying attacks and then implementing appropriate countermeasures. In this section, we highlight the contributions and limitations of related works relevant to this research topic.

### 2.1. Intrusion Detection in IoT Using ML and FL

To minimize the threat of intrusions and other cyberattacks, centralized machine learning models work well but have vulnerabilities like single points of failure (SPoFs). To address this, researchers have proposed FL-based IDSs using conventional and advanced ML techniques.

Zhao et al. [21] employed Long Short-Term Memory (LSTM) and Recurrent Neural Networks (RNNs) within an FL framework, achieving high accuracy and F1-Scores. Driss et al. [22] developed an FL-based IDSs for vehicular sensor networks (VSNs) using Gated Recurrent Units (GRUs) and ensemble methods, demonstrating high performance in attack detection. Khan et al. [23] proposed a Stackelberg game-based method to improve FL communication among edge servers, highlighting its potential for handling large datasets efficiently. Nguyen et al. [24] introduced a self-learning anomaly detection model using FL, GRUs, and LSTM, which continuously monitors network traffic for anomalies. Du et al. [25] addressed security in vehicular IoT devices with a Multi-access Edge Computing (MEC) solution, enhancing system performance and security. Mothukuri et al. [26] presented an FL-based anomaly detection solution using GRU algorithms, which outperformed traditional centralized ML approaches in accuracy and data privacy protection.

Recent advancements in machine learning have introduced Graph Neural Networks (GNNs) and temporal graphs as powerful tools for modeling complex data relationships and temporal dynamics. In the context of the IoT and IoMT, these approaches can significantly enhance IDSs by capturing intricate dependencies and evolving patterns of network traffic and device interactions. GNNs have shown significant promise in modeling relational data and capturing dependencies in graph-structured data. IoT devices and their interactions can naturally be represented as a graph, where nodes represent devices and edges represent communication links or data flows. Studies such as those by Wu et al. [27] and Deng et al. [28] demonstrate the potential of GNNs in capturing complex interactions for anomaly detection. GNNs can effectively capture these interactions, making them suitable for tasks like anomaly detection, where understanding the relationship between devices can lead to better detection of coordinated attacks. Additionally, GNNs are inherently scalable and can handle large-scale networks, which is critical in IoT environments with numerous interconnected devices [29]. Integrating GNNs within our Federated Learning framework could enhance detection capabilities by leveraging the relational data inherent in IoT networks. Kong et al. [30] presented a robust framework that can handle the complexities of graph-structured data. They used contrastive self-supervised learning to improve the quality of data representations, leading to more accurate anomaly detection.

Temporal graphs extend the concept of static graphs by incorporating time as a dimension, capturing the evolution of the network over time. This is particularly useful in dynamic environments like the IoMT, where device interactions and data flows change frequently. Approaches like SigTran focus on the temporal aspects of the graphs, capturing changes and trends over time. In intrusion detection, SigTran can identify temporal patterns in attack behaviors, offering a more dynamic and responsive detection mechanism [31]. Similarly, TGBASE [32] leverages temporal graph structures to enhance anomaly detection by considering the temporal evolution of data, which is critical in identifying sophisticated and evolving cyber threats. Incorporating temporal graph methodologies into our FL-based IDSs can provide a more robust framework, enhancing the detection of time-based anomalies and evolving threats, thereby complementing our current focus on model accuracy and robustness.

### 2.2. Intrusion Detection Using Blockchain and FL

By enabling participants to transact and share information while preserving a level of trust, integrity, and greater transparency, blockchain has been used in a variety of domains to facilitate trust and data privacy. Therefore, blockchain technology offers applications that go beyond those related to finance and economic systems [13]. On cloud and IoT networks, IDSs and blockchain can be combined to identify cyberattacks and protect sensitive data.

In their study, Alexopoulos et al. [33] explored combining CIDSs and blockchain to increase trustworthiness and accountability. Rashid et al. [34] proposed a decentralized and secure data traceability framework to enhance data privacy and combat deepfakes. Zaabar et al. [35] introduced a blockchain-based FL architecture for IoMT environments to identify malicious network traffic, replacing traditional central servers with a Hyperledger Fabric channel.

Casado-Vara et al. [36] presented a hybrid IoT architecture using blockchain for decentralized data management and edge computing. Alkadi et al. [37] used Bidirectional Long Short-Term Memory (BiLSTM) and smart contracts to secure decentralized IDSs, demonstrating superior performance. Kumar et al. [38] used fog computing for a decentralized IDS to detect DDoS attacks, showing that Random Forest outperforms other methods in multi-attack detection, while XGBoost excels in binary attack detection. Sindhusaranya et al. [39] proposed FL-BEPP for fraud prevention and security in the IoMT framework, focusing on efficient identification and privacy protection across fog and cloud nodes.

Golomb et al. [40] introduced CIoTA, an IoT-based distributed anomaly detection framework using blockchain to enhance network and device security. Lakhan et al. [41] developed FL-BETS, a blockchain-enabled task scheduling framework for healthcare applications, ensuring fraud detection and privacy preservation. Dey et al. [42] proposed an intelligent software agent using machine learning and game theory to identify malicious transactions and estimate attack probabilities.

Lian et al. [43] introduced a blockchain-based data sharing scheme that can significantly improve the accuracy of the model without compromising user privacy to address the issue of poor training performance on non-independent identically distributed (non-IID) data. To further boost the effectiveness of the system, they also created a client selection mechanism. Eskandari et al. [44] proposed a distributed FL approach using blockchain to address poisoning attacks, ensuring compromised clients are accurately recognized.

Faheem et al. provided an innovative solution combining Federated Learning and blockchain to enhance security and privacy in IoT networks. It offers decentralized storage solutions and smart contracts for efficient data management. They also demonstrated a practical application of blockchain for secure data traceability and privacy in multimedia content, addressing issues like deepfakes and cyberattacks. In another work, these authors presented a Federated Learning-based architecture leveraging blockchain for secure and efficient intrusion detection in IoMT environments. It replaces traditional central servers with a Hyperledger Fabric channel. These presented methods [45,46,47] can be used in multimedia applications, ensuring secure data handling and preventing unauthorized alterations, which is crucial for media industries and online content platforms. Table 1 summarizes the strengths, weaknesses, and areas of applications of related works.

In our research, we concentrated on creating IDSs for the Internet of Medical Things (IoMT) by leveraging blockchain and Federated Learning. While existing studies can detect breaches, their performance in data storage and accuracy falls short. To enhance intrusion detection in IoT networks, we implemented Federated Learning and leveraged blockchain technology to safeguard the privacy of IoMT data.

## 3. Proposed Method

The training data from the devices to be simulated are utilized to develop the model in traditional anomaly detection scenarios. However, this method faces challenges in the IoMT paradigm. Most IoMT devices often have a single purpose and a small number of features; thus, they do not generate a lot of data. It can take some time to gather enough data to train a consistent model, which makes it challenging to train a model entirely on data obtained from a user’s local network. This calls for a technique that combines training data from several users into a single training dataset to expedite the learning of a reliable model. Instead of sending data to a central entity, such as a fully centralized learning model, each client (which can be a local WiFi router connected to several IoT devices) trains a local model using training data that are easily accessible locally in an FL setting. This section explains how our suggested method works. Data storage and protection are handled by the blockchain-based aggregation server alone, with the aid of decentralized storage systems (a combination of IPFSs and MongoDB). To prevent any form of unauthorized transaction, the FL distributor is thoroughly governed and maintained by the smart contracts. As a result, the security of the blockchain method forms the basis of the security of our system.

### 3.1. Framework

**Figure 2:**
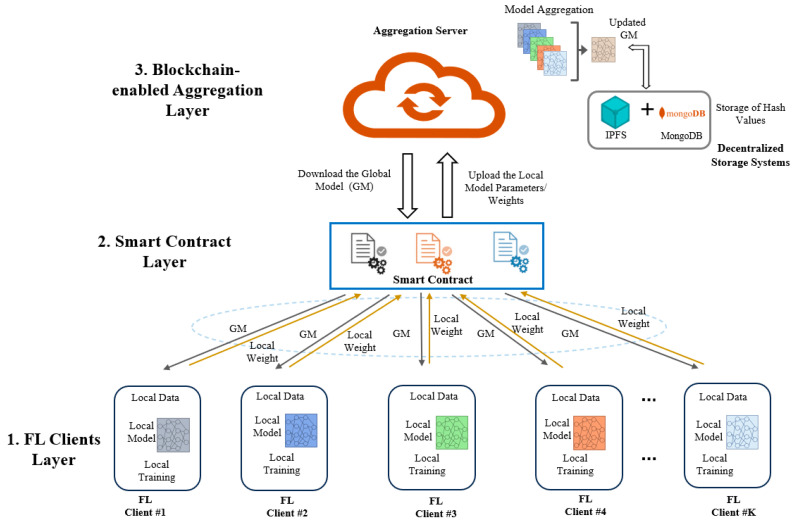
Proposed BC-enabled FL architecture for IoMT intrusion detection.

We propose a Federated Learning-based approach for ML and blockchain-enabled IoMT network intrusion detection. Figure 2 depicts the framework, where several devices are put in various locations and linked to the network. We describe in detail each operation that was undertaken to implement the strategies of the proposed method in the following subsections. Our proposed FL model is divided into three layers, as follows:

- FL Client Layer: incorporates local training capabilities, allowing ML models to run directly on end devices. This layer ensures data privacy and security by keeping data local.
- Smart Contract Layer: manages interactions between clients and the blockchain-enabled aggregation server using smart contracts, ensuring secure and transparent operations.
- Blockchain Layer: acts as a secure storage and aggregation server, utilizing decentralized storage systems like IPFSs and MongoDB for efficient data management and redundancy.

#### 3.1.1. FL Client Layer for Local Training

This layer enables IoMT devices to train ML models locally, removing the need for centralized servers. Each BC-enabled *k* FL client ((here, *i* ∊ [1, …, *N*]) at the local end trains the data from their own IoMT devices with the local models provided by the server, and the IDS at the client end operates to recognize any unwanted attacks. Using local training, parameter adjustment, and improved inference techniques, such on-device intelligent learning enables the independence of local intrusion detection. Additionally, this layer is responsible for ensuring the security of the local data. There is extremely little possibility of any kind of falsification once we distribute clients with BC because local transactions are time-stamped and immutable. Local training allows for real-time analysis and decision-making at the point of care, reducing latency, bandwidth usage, and dependency on external resources.

Here are the key components of this layer:

- **Local model training:** Each FL client (IoMT device) collects data and trains a local model using ML algorithms, such as CNNs, and LSTM, which were initially distributed as a global model (GM) by the server. This helps in detecting potential intrusions by analyzing patterns in the device’s data.
- **Data privacy and security:** Training happens entirely on the local IoMT devices, which means raw data are never sent to external servers. This ensures privacy and minimizes the risk of data breaches.
- **Model update generation:** After training, IoMT devices create local model updates that represent learned insights. These updates are shared, but the raw data are not, preserving privacy.
- **Blockchain integration:** The FL Client Layer interacts with the blockchain to record and verify these model updates. This allows for transparent auditing and documentation of each client’s contributions.
- **Privacy-preserving communication:** Secure communication mechanisms are used to ensure the integrity and confidentiality of the model updates during transmission.

#### 3.1.2. Smart Contract Layer for FL Distribution

Smart contracts enforce data privacy and security measures, ensuring that sensitive information shared during FL tasks is protected and only accessible to authorized parties. In this layer, the clients collaborate with an aggregation server to aggregate local models and build a more robust IDS with optimal settings. The FL distributor (for example, a security gateway) uses smart contracts to handle all interactions between the clients and the blockchain-enabled aggregation server. This layer manages the distribution and aggregation of local models using smart contracts to ensure data privacy and security:

- **Record storage contract:** This smart contract defines a structured approach to storing records within the blockchain. It includes a struct named “Record” that encapsulates essential information such as the client ID, hash of the record, and timestamp.
- **Storage mapping:** The contract utilizes a mapping data structure to associate each record with a unique identifier (ID). The mapping allows efficient retrieval of records based on their IDs.
- **Event emission:** Whenever a new record is stored using the “storeRecord” function, the contract emits a “RecordStored” event. This event provides a way for external parties to monitor and react to changes in the contract state.
- ***StoreRecord function*:** This function enables the addition of new records to the storage. It receives inputs such as the record ID, client ID, and hash of the record. Before storing a new record, the function checks that the provided ID is not already in use to prevent overwriting existing records.
- ***GetRecord function*:** The “getRecord” function allows clients to retrieve specific records from the storage based on their IDs. It takes the ID of the record as input and returns the corresponding client ID, hash, and timestamp. If the requested record does not exist, the function throws an error indicating that the record was not found.

#### 3.1.3. Blockchain as Secure Storage and FL Aggregation

The blockchain-enabled aggregation server possesses significant computing power, allowing it to aggregate parameters shared by participating clients for global aggregation. This layer involves the aggregation server and decentralized storage systems to manage and update the global model:

- **Aggregation and Global Model Update**: The aggregation server gathers all local weights before training and updates the global model using the aggregation algorithm. Devices or nodes interested in participating in the FL process register with the FL aggregation server. It deploys smart contracts on the blockchain network to manage FL tasks, including participant registration, model aggregation, parameter updates, and reward distribution.
- **Decentralized Storage Systems**: The layer incorporates decentralized storage systems comprising the InterPlanetary File System (IPFS) and MongoDB. IPFSs enable the distributed storage and retrieval of model updates across a network of nodes, ensuring redundancy and fault tolerance. MongoDB complements IPFSs by providing a structured database for indexing and querying model updates based on metadata attributes such as timestamp, client ID, and model version. By leveraging decentralized storage solutions, the FL system mitigates the risk of data loss, censorship, or single points of failure inherent in centralized storage architectures. This distributed storage approach enhances data availability, accessibility, and resilience, fostering trust and reliability in the Federated Learning process.
- **RESTful API**: The RESTful API facilitates the seamless upload and download of local weights, as well as the upload of the global model. Through designated endpoints, clients can securely upload their local weights, download the latest model updates, and contribute to the enhancement of the global model. The API ensures efficient communication between clients and the server, enabling real-time interaction with the blockchain network. This streamlined process empowers clients to actively participate in the collaborative learning paradigm, contributing to the continuous improvement of the IDS while maintaining data privacy and integrity.

### 3.2. Adversary Model and Assumptions

In an IoMT network, an adversary *M* can be either internal or external. External adversaries typically conduct web-based attacks that affect digitally equipped devices, introducing malicious content into databases or stealing personal information. Conversely, a compromised IoMT device or another networked device could act as an insider threat if it continues to operate within the network. IoMT malware is capable of identifying and exploiting vulnerabilities in IoMT systems and devices, using less secure devices as platforms for launching cyberattacks. The manufacturing of IoMT products may be exploited by malware, traffic congestion, commercial fraud caused by the introduction of fraudulent product data, compromise of inadequate quality and control systems, XSS and SQL injections, and DDoS, which are only a few examples of IoMT network-based attacks. There are also additional attacks that target IoMT protocol. A few instances of IoMT protocol-based attacks include the following: compromising surveillance, GPS spoofing, and manipulating farm machinery to delay or make poor decisions.

In our work, we have made some assumptions for our design to work. They are:

- **Participant availability:** We assume that the participants in the FL network are consistently available for training and sharing their local models. This assumption ensures the timely aggregation of model updates and prevents delays in the learning process.
- **Secure communication between FL clients and IoMT devices:** For our Intrusion Detection System to operate effectively, it needs to communicate with participating Federated Learning (FL) clients and IoMT devices. We assume that these communication channels are secure and protected from any potential threats.
- **Absence of malicious IoMT devices:** A new IoMT product may already have security flaws when an IoMT provider first introduces it. These products should not be compromised or corrupted in any way during initial use. This will allow the system to generate only permitted interactions before an adversary M identifies and exploits any flaws, allowing our model to learn from the safe patterns.

### 3.3. Federated Learning Algorithm and Procedure

As previously mentioned, in our Federated Learning (FL) model, all *N* FL clients train a local model using their private datasets with a globally shared model provided by the FL distributor rather than training and evaluating on a central server. They then transfer the knowledge acquired from these local trainings to the aggregation server via a secure SSL/TSL communication channel (e.g., gRPC channel [48]). The aggregation server combines this information to produce an enhanced global model with optimal parameters. Here, *ω* represents the initial weights and *R* denotes the number of FL rounds. The model weights can be updated using the formula in Equation (1), based on the FedAvg [10] algorithm, when each local client’s weight is transmitted to the aggregation server during communication round *t*. *N* indicates how many local instances of client *i* there are.

(1) $$\omega_{t+1}=\overset{N}{\underset{i=1}{\sum }}\frac{D_{i}}{D}{\omega_{i}}^{\left(t+1\right)}$$

where D represents the total volume of all client datasets and $D_{i}$ denotes the size of each client dataset. This approach is used to calculate the weighted average of the new weight file. Once the updated weight $\omega_{t+1}$ is loaded, the model is aggregated to form the global model. Subsequently, each client receives and processes the updated global model continually.

To enhance the FedAvg algorithm, we incorporated a divergence estimation technique (the Kullback–Leibler (KL) divergence) and adaptive weight calculation. This improves the model’s accuracy and robustness against adversarial attacks.

**Divergence estimation:** Divergence estimation involves quantifying the dissimilarity and discrepancy between the data distribution of each client and the global model distribution. It provides insights into how representative each client’s data are compared to the overall data distribution captured by the global model. For divergence estimation in Federated Learning settings, we used the Kullback–Leibler (KL) divergence. The KL divergence measures the relative entropy between two probability distributions and provides a quantification of the information lost when using one distribution to approximate the other. It is represented by Equation (2).

(2) $$D_{KL}(P_{i}||P_{t})=\underset{i}{\sum }P_{i}\left(i\right)\log\left(\frac{P_{i}\left(i\right)}{P_{t}\left(i\right)}\right)$$

Here, this equation calculates the KL divergence between the local model *P_i_* and the global model *P_t_*. The sum is over all possible outcomes *i*.

**Weight calculation:** Once the divergence between each client’s data distribution and the global model distribution is estimated, the next step is to calculate importance weights for each client. These weights reflect the significance or relevance of each client’s data about the global model. For weight calculation, we used the inverse of the estimated divergence as the importance weight. This approach ensures that clients with data distributions more similar to the global model contribute more to the aggregation process, while those with divergent data distributions contribute less.

*Adaptive Weight Calculation:*

To adaptively adjust the contribution of each client’s local model during aggregation, we use the KL divergence to compute the weights:

(3) $$\alpha_{i}^{\left(t\right)}=\frac{1}{1+\lambda D_{KL}(P_{i}||P_{t})}$$

This equation calculates the adaptive weight $\alpha_{i}^{\left(t\right)}$ for client *i* at round *t*. The weight is inversely proportional to the KL divergence. The parameter *λ* controls the sensitivity of the weight adjustment.

*Global Model Aggregation:*

The global model is updated by aggregating the local models using the adaptive weights:

(4) $$\omega_{avg}^{\left(t+1\right)}=\frac{\sum_{i=1}^{N}\alpha_{i}^{\left(t\right)}\omega_{i}^{\left(t+1\right)}}{\sum_{i=1}^{N}\alpha_{i}^{\left(t\right)}}$$

(5) $$\omega_{t+1}=\omega_{t}+\eta_{q}\omega_{avg}^{\left(t+1\right)}$$

These equations update the global model *ω_t_*_+1_ at round *t* + 1 by taking a weighted average of the local models $\omega_{i}^{\left(t+1\right)}$. In our implementation, the adaptive weights $\alpha_{i}^{\left(t\right)}$ are computed considering the significance of each client’s data, which naturally reflect the computational and energy capabilities of IoMT devices. By emphasizing data quality and relevance, the aggregation process remains efficient, even in resource-constrained environments.

*Adaptive Learning Rate:*

To further enhance the robustness and accuracy, the learning rate is adjusted based on the KL divergence:

(6) $$\eta_{i}^{\left(t\right)}=\eta \times \frac{1}{1+\lambda D_{KL}(P_{i}||P_{t})}$$

This equation calculates an adaptive learning rate $\eta_{i}^{\left(t\right)}$ for client *i* at round *t*. The base learning rate *η* is scaled by a factor that is inversely proportional to the KL divergence. In the context of the IoMT, the KL divergence effectively captures the distribution differences, ensuring that devices with more representative data have a greater influence on the global model. This method inherently recognizes the computational and energy limitations by focusing on the contribution of data quality over sheer volume or computational power.

Algorithm 1 represents an algorithm that adjusts the aggregation process based on the characteristics of the participating clients and their data distributions. Divergence estimation allows us to quantify the dissimilarity between each client’s data distribution and the global model distribution. By incorporating divergence estimation and weight calculation, the Federated Learning system becomes more robust and adaptable to changes in the data distribution and client dynamics over time.
**Algorithm 1.** Adaptive Federated Learning Algorithm with Divergence Estimation and Weight Calculation.**Input:** Global model parameters *ω_t_* Local datasets for each client *D_i_* Local learning rate schedule for each client *η_i_*(*t*) **Output:** Updated global model parameters *ω_t_*_+1_ **//Client-Side:** 1: **for** each client *i* in parallel **do** 2:   **for** each epoch *e*
**do** 3:   Train local copy of *ω_t_* on *D_i_* using *η_i_*(*t*) 4:   Obtain $\omega_{i}^{\left(t+1\right)}$ 5:   **end for** 6:     Calculate local performance metrics on $\omega_{i}^{\left(t+1\right)}$ and *D_i_* 7:     Estimate divergence between *P_i_* (client’s data distribution) and *P_t_* (global model distribution) using        the Kullback–Leibler (KL) divergence (Equation (2)): 8:      **if** divergence is acceptable **then** 9:       Calculate importance weight $\alpha_{i}^{\left(t\right)}$ based on divergence estimation: 10:    **else** 11:      Adjust learning rate *η_i_*(*t*) and retrain local model 12: **end if** 13: **end for** **//Server-Side:** 14: Sample clients with probability proportional to $\alpha_{i}^{\left(t\right)}$ 15: Collect $\omega_{i}^{\left(t+1\right)}\;$ from selected clients 16: Aggregate updates using weighted average (Equation (4)) with $\alpha_{i}^{\left(t\right)}$ as weights: 17: Update the global model using (Equation (5)). 18: **if** the global model converges **then** 19:     Broadcast *ω_t_*_+1_ to all clients 20: **else** 21:     Repeat the process for the next round 22: **end if**   Updated global model parameters *ω_t_*_+1_

### 3.4. ML Classifiers for Intrusion Detection

The rapid advancement of machine learning techniques and applications has opened up new growth opportunities for IDS solutions. Neural network-based methods have proven highly effective in achieving better data representations for successful modeling. Although neural networks come in various forms, they all share four fundamental components: neurons, weights, biases, and functions. A common application of neural networks is to establish a relationship between an input *x* and an output *y* such that *y* = *f*(*x*,θ), where θ is the parameter vector. To maintain simplicity, we limited the number of classifiers for intrusion detection, with each classifier training on the given dataset. We employed two classifiers: Bidirectional Long Short-Term Memory (BiLSTM) and Convolutional Neural Networks (CNNs).

#### 3.4.1. Convolutional Neural Network (CNN)

CNNs are responsible for the analysis of data presented as numerous arrays. Convolutional feature extractors are used to process a group of teachable filters, which make up the first layers of this approach. The applied filters create a sliding window that allows users to view each piece of input data. In this context, the stride refers to the overlapped distance, and the results are referred to as feature maps. In a CNN layer, which is used to create various feature maps, convolutional kernels are included. The feature map of the layer below shows a relationship between a neuron and neighboring neuron regions. The kernel needs to be dispersed among all input spatial locations to produce a feature map. One or more completely linked layers are used to complete the classification once the convolutional and pooling layers have been created [49]. Convolutional operations on input feature maps and convolutional layers are represented in CNN architectures by Equation (7).

(7) $$h_{j}^{\left(n\right)}=\overset{K}{\underset{k=1}{\sum }}h_{k}^{\left(n-1\right)}*w_{kJ}^{\left(n\right)}+b_{kJ}^{\left(n\right)}$$

where ∗ is the convolution operator and $h_{j}^{\left(n\right)}$ represents the *j*th feature map in the *n*th hidden layer. The *k*th channel of the (*n* − 1)th hidden layer is denoted by $h_{k}^{\left(n-1\right)}$, while the weights of the *k*th channel in the *j*th filter of the *n*th layer are represented by $w_{kJ}^{\left(n\right)}$ and the pertinent bias term is denoted by $b_{kJ}^{\left(n\right)}$.

**Figure 3:**
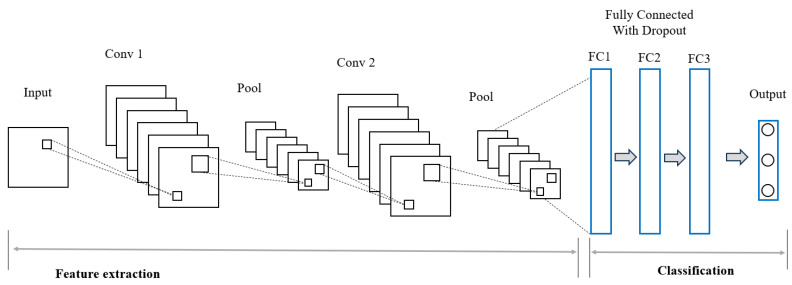
CNN model used as an ML classifier at the local client’s end.

Figure 3 shows the CNN model we used as an ML classifier at the local client’s end. This model has two convolutional layers, three fully connected layers, and one dropout layer.

In our Federated Learning Intrusion Detection System (FL IDS), we employ an Adaptive Max Pooling-based CNN model to enhance the network’s ability to handle inputs of varying sizes while ensuring consistent output dimensions. This approach significantly improves the model’s robustness and efficiency.

The Adaptive Max Pooling Layer reduces the spatial dimensions of the feature maps to a fixed size:

(8) $$\left(i,j\right)=\underset{0\leq m<k_{h},0\leq n<k_{w}}{max}h_{j}^{\left(i\right)}\left(s_{h}\cdot i+m,s_{w}\cdot j+n\right)$$

where *y*(*i*,*j*) is the pooled output, *k_h_*, and *k_w_* are the kernel sizes, *s_h_* and *s_w_* are the strides, and $h_{j}^{\left(i\right)}$ is the input feature map from the previous layer.

The integration of Adaptive Max Pooling Layers offers several benefits:

- Flexibility: can handle varying input sizes while producing consistent output dimensions.
- Efficiency: reduces computational load by dynamically adjusting pooling operations.
- Robustness: maintains spatial hierarchies and patterns in the input data.

By incorporating Adaptive Max Pooling layers, our CNN model becomes more robust, flexible, and efficient, thereby improving the performance of Federated Learning.

#### 3.4.2. Bidirectional Long Short-Term Memory (BiLSTM)

Long Short-Term Memory (LSTM) is a special sort of RNN model. The cell state addition in the LSTM network sets it apart from the conventional RNN. The cell state with time t will be passed to the following cell, which stands for long-term memory. Each cell autonomously chooses whether to forget information throughout the calculation of the cell sequence, allowing it to keep prior information for a considerable amount of time [50]. The following equation (Equation (9)) is applied to create the LSTM hidden layer at time step *t*:

(9) $$i_{t}=\sigma \left(W_{x,i}x_{t}+W_{h,i}h_{t-1}+W_{c,i}c_{t-1}+b_{i}\right)\;f_{t}=\sigma \left(W_{x,f}x_{t}+W_{h,f}h_{t-1}+W_{c,f}c_{t-1}+b_{f}\right)\;c_{t}=f_{t}⊙c_{t-1}+i_{t}⊙\tanh\left(W_{x,c}x_{t}+W_{h,c}h_{t-1}+b_{c}\right)\;o_{t}=\sigma \left(W_{x,o}x_{t}+W_{h,o}h_{t-1}+W_{c,o}c_{t}+b_{o}\right)\;h_{t}=o_{t}⊙\tanh\left(c_{t}\right)\;y_{t}=\mathrm{SoftMax}\left(Wh_{t}+b\right)$$

where $i_{t}$ stands for the input gates, $f_{t}$ represents the forget gates, $c_{t}$ represents the memory cells, $o_{t}$ represents the hidden output, and $y_{t}$ represents the output layer.

**Figure 4:**
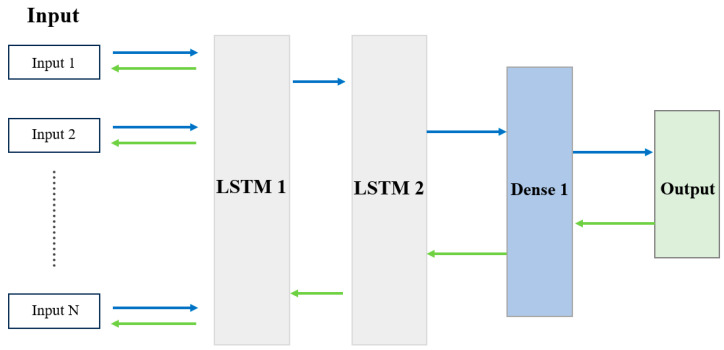
BiLSTM model used as an ML classifier at the local client’s end.

To improve sequence prediction, allow the LSTM model to learn the input sequence both forward and backward and combine the two methods of understanding it. Bidirectional LSTM enhances a model’s ability to predict future events. BiLSTM trains two LSTMs instead of one when the input sequence’s time steps are all known. The first is trained on the original input sequence, while the second is trained on a copy of the original sequence with the time steps switched. Providing more context can help the network learn the problem faster and more thoroughly. Our hypothesis implies that a BiLSTM with the simplest structure outperforms a conventional LSTM in terms of accuracy. We chose the simplest structure for optimal performance as it takes less time to construct the model and requires less memory for computation. Figure 4 is a detailed illustration.

To further enhance the performance of the BiLSTM model, we incorporate an attention mechanism and a residual connection mechanism:

*(a) Attention mechanism:* The attention mechanism allows the model to focus on relevant parts of the input sequence when making predictions. The attention weights $a_{tj}$ are calculated as follows:

(10) $$a_{tj}=\frac{\exp\left(e_{tj}\right)}{\sum_{k=1}^{T}\exp\left(e_{tk}\right)}$$

where $e_{t}=v^{T}\tan h\left(W_{h}h_{t}+W_{s}s_{t-1}+b\right)$.

Here, $a_{tj}$ represents the attention weight at time step t, which determines the importance of the hidden state $h_{t}$ for the current prediction. The score $e_{t}$ is computed as a dot product of a learnable parameter vector *v* and the non-linear transformation of the hidden state $h_{t}$ and the previous state $s_{t-1}$. The weight matrices $W_{h}$ and $W_{s}$ transform the hidden state and the previous state, respectively, while b is a bias term added to the linear combination. The hyperbolic tangent function tanh introduces non-linearity, ensuring the model captures complex patterns in the data.

The context vector $c_{t}$ is then computed by Equation (11).

(11) $$c_{t}=\overset{T}{\underset{j=1}{\sum }}a_{tj}h_{t}$$

The final output $y_{t}$ is given by:

(12) $$y_{t}=\mathrm{SoftMax}\left(W_{y}\left[c_{t};\;h_{t}\right]+b_{y}\right)$$

where $\left[c_{t};\;h_{t}\right]$ denotes the concatenation of the context vector and the BiLSTM’s hidden state, respectively, and $W_{y}$ and $b_{y}$ are learnable parameters.

*(b) Residual connection mechanism:* To further improve the model’s performance and training stability, we introduce residual connections, which help mitigate the vanishing gradient problem and allow for deeper networks. The residual connection for the BiLSTM output is given by:

(13) $$h_{t}^{res}=h_{t}+f\left(h_{t}\right)$$

where $f\left(h_{t}\right)$ is a learnable transformation (e.g., a fully connected layer) applied to the hidden state $h_{t}$. The final output *y_t_* then incorporates this residual connection:

(14) $$y_{t}=\mathrm{SoftMax}\left(W_{y}\left[c_{t};\;h_{t}^{res}\right]+b_{y}\right)$$

By integrating these mechanisms, our BiLSTM model becomes more robust, accurate, and efficient in the context of FL-based IDSs.

## 4. Experiments, Results, and Discussion

This section offers a thorough analysis of performance as well as experimental information for our suggested approach.

### 4.1. Experimental Setup

The primary programming language we used for our experiments was Python 3 and Node.js. Multi-dimensional arrays and matrices, along with NumPy and several other well-known libraries, are used to achieve our method. Pandas provides robust capabilities for analyzing and manipulating data structures. Additionally, ML and DL are performed using TensorFlow and Keras. Also, Scikit-learn offers a wide variety of supervised and unsupervised ML method implementations. Additionally, we oversampled minority classes using SMOTE [51] to improve the overall model effectiveness.

The Ethereum smart contracts are created using the Solidity programming language [52] and are assembled and tested using the Remix IDE [53]. We also developed a local blockchain by using Node.Js. We used Express.js to build our RESTful API, which was responsible for the upload and download of the models. Later, in this paper, we shall discuss the characteristics of these smart contracts in more detail.

### 4.2. Datasets and Pre-Processing

Selecting the appropriate dataset is crucial for IoT networks as they require data for both training and testing Intrusion Detection Systems (IDSs). Due to the absence of FL-specific datasets, our experiment utilized a pre-existing publicly available dataset, which we divided to simulate data federations.

For industrial IoT (IIoT) and IoT applications, the Edge-IIoTset [54] dataset is widely used in cybersecurity research. The data were produced by a variety of IoT devices, including heart rate sensors, fire sensor systems, humidity and temperature sensors, etc. The testbed was attacked in 14 distinct ways, including DDoS attacks, Backdoor attacks, man-in-the-middle (MITM) attacks, ransomware, and password attacks. The Edge-IIoT dataset contained 1,909,671 samples, 15 classes, and 61 different features. Sets of 1,527,736 and 381,935 data points for training and testing were available. As a first step, we aggregated the data and eliminated any duplicates or missing information, such as “NAN” (Not a Number) and “INF” (Infinite Value). Further flow characteristics were then removed, including tcp.payload, tcp.options, tcp.len, tcp.flags.ack, tcp.flags, tcp.connection.rst, tcp.connection.fin, tcp.checksum, tcp.ack raw, tcp.ack, and arp.dst.proto ipv4. Following data pre-processing (cleaning and splitting), we chose an 80/20 ratio for the data for ML models to train and test the datasets.

The ToN-IoT dataset [55], which consists of numerous attack scenarios, was utilized. Since it is the first IoT network intrusion dataset to incorporate data from four disparate sources (pcap files, Bro logs, sensor data, and OS logs), special care was taken to ensure that a wide range of devices and attack types were included. Nine different cyberattack types, including password cracking attacks, ransomware, data injection, scanning, denial-of-service attacks, cross-site scripting (XSS), and man-in-the-middle (MITM), were launched against different IIoT and IoT sensors throughout the IIoT network. Seven IoT and IIoT sensors (such as weather, temperature, and Modbus sensors) were used to collect their telemetry data, and two smartphones and a smart TV were used to log network traffic. The generated data were saved in log and CSV files. The dataset consisted of 22,339,021 total flows, of which 796,380 (3.56%) were benign flows and 21,542,641 (96.44%) were attack samples. To simulate a real-world execution of current production IoT/IIoT networks, the TON_IoT dataset was designed using interacting network elements and IoT/IIoT systems with the three layers of Edge, Fog, and Cloud. The NSX-VMware platform was employed to enable the management of the interplay among these three layers through the use of Software-Defined Network (SDN) and Network Function Virtualization (NFV) technologies.

To assess the effectiveness of FL, we ran several experiments with 5 to 20 clients participating in model training. According to our findings, the local model for every client should undergo training for a total of 20 epochs and 50 FL rounds. While creating the federated model, we looked at how effective the system was for various client counts. The deployment dataset’s training data were distributed to each client, from which a random selection was made. By distributing the complete training dataset among 5, 10, and 20 clients, we created three federated models to assess this potential loss in accuracy. We then compared these models to a centralized model.

### 4.3. Evaluation Metric

A confusion matrix [56] was used to define True Positive (TP), False Positive (FP), False Negative (FN), and True Negative (TN) for multiple classes.

- True Positive (TP): the number of samples accurately identified as attacks out of all samples.
- False Positive (FP): the number of benign samples mistakenly labeled as attacks.
- True Negative (TN): the number of neutral samples correctly classified as normal.
- False Negative (FN): the number of attack samples incorrectly categorized as normal.

- Accuracy (Acc): One measurement for assessing the classification model’s performance is accuracy. The accuracy measurement is represented by Equation (15). It is a measure of the proportion of correctly classified inputs among all inputs.

(15) $$\mathrm{Acc}=\frac{\mathrm{TP}+\mathrm{TN}}{\mathrm{TP}+\mathrm{TN}+\mathrm{FP}+\mathrm{FN}}$$

- Precision (Pre): Precision is the ability to make accurate predictions. It is a measurement of the ratio of the model’s claimed positives to the number of actual positives and is represented by Equation (16).

(16) $$\mathrm{Pre}=\frac{\mathrm{TP}}{\mathrm{TP}+\mathrm{FP}}$$

- Recall (Detection Rate): The recall is often referred to as the real positive rate, which measures the proportion of positives in model claims to the actual number of positives found across the data. Equation (17) depicts the recall measurement for a model.

(17) $$\mathrm{Recall}\;\left(\mathrm{Detection}\;\mathrm{Rate}\right)=\frac{\mathrm{TP}}{\mathrm{TP}+\mathrm{FN}}$$

- F1-Score: A model’s performance can also be evaluated using the F1-Score. It represents a model’s weighted average of recall and precision and is given by Equation (18).

(18) $$F_{1}\;\mathrm{Score}=2\cdot \frac{\mathrm{Precision}\cdot \mathrm{Recall}}{\mathrm{Precision}+\mathrm{Recall}}$$

### 4.4. Performance Evaluation

The experiment using centralized training and the efficiency of our proposed FL-based model for intrusion detection that used the Edge-IIoTset dataset are discussed in this section.

#### 4.4.1. Intrusion Detection Using Centralized Methods

We first used CNNs and BiLSTM, two established centralized ML methods for cyberattack detection, to assess the proposed dataset’s accuracy.

Table 2 displays the outcomes of ML techniques for a centralized model based on precision, recall, and F1-Score, which measures how well the model distinguishes between benign and attack classes from the dataset.

**Edge-IIoT dataset:**

For normal or benign behavior, the CNN model achieves a precision of 0.94, recall of 0.92, and F1-Score of 0.94, while the BiLSTM model shows similar performance with a precision of 0.92, recall of 0.91, and F1-Score of 0.91. Both models perform well in detecting backdoor attacks, with precision, recall, and F1-Score ranging from 0.93 to 0.94. For DDoS attacks (HTTP- and ICMP-based), the models achieve precision, recall, and F1-Scores above 0.87. However, for TCP- and UDP-based DDoS attacks, the performance drops, especially for UDP attacks, with scores around 0.65. The models’ performance for various other attacks, including fingerprinting, MITM, and ransomware, varies, with a precision, recall, and F1-Score ranging from 0.68 to 0.95 for both models.

**TON_IoT dataset:**

Both models achieved a high precision, recall, and F1-Score for detecting normal behavior, with values ranging from 0.91 to 0.94. For DDoS, injection, password, backdoor, ransomware, XSS, and scanning attacks, the CNN and BiLSTM models showed consistent performance across different attack types, with a precision, recall, and F1-Score above 0.90 for most classes.

#### 4.4.2. Intrusion Detection using Blockchain-Enabled FL Method

We first used CNNs and BiLSTM, two established centralized ML methods for cyberattack detection, to assess the proposed dataset’s accuracy.

In our model’s FL tests, we deployed three sets of clients: *K*, *K* = 10 (first set), *K* = 15 (second set), and *K* = 20 (third set).

Table 3 presents a comparison of accuracy outcomes for all global models, as well as the best and worst clients, at the 1st and 50th FL rounds using a distributed dataset. The data indicate that as the training rounds progress, the performance of all classes improves, and the gap between the best and worst clients narrows. Initially, in the 1st FL round, there is a significant difference in accuracy between the best and worst clients. However, this difference becomes less pronounced by the 50th round.

**Edge-IIoT dataset:**

CNN: For *K* = 10 clients, the best-performing client achieved an accuracy of 53.00% in the 1st FL round, while the worst-performing client achieved an accuracy of 37.94%. The global accuracy after the 50th FL round improved to 85.31%. Similarly, for *K* = 15 and *K* = 20 clients, the accuracy ranged from 45.86% to 46.29% in the 1st FL round, with the global accuracy improving to approximately 85% after the 50th FL round.

LSTM: The LSTM model shows varying performance across different numbers of clients. For *K* = 10 clients, the best- and worst-performing clients achieved accuracies of 41.99% and 44.42% in the 1st FL round, respectively. The performance of all the clients improved with the increase in rounds. The global accuracy improved to 83.24% after the 50th FL round. Similar trends are observed for *K* = 15 and *K* = 20 clients, with accuracies ranging from 47.72% to 48.95% in the 1st FL round and global accuracies improving to approximately 83% after the 50th FL round.

**TON_IoT dataset:**

CNN: For *K* = 10 clients, the best-performing client achieved an accuracy of 57.00% in the 1st FL round, while the worst-performing client achieved an accuracy of 40.94%. The global accuracy after the 50th FL round improved to 87.95%. Similarly, for *K* = 15 and *K* = 20 clients, the accuracy ranged from 47.86% to 48.29% in the 1st FL round, with the global accuracy improving to approximately 87% after the 50th FL round.

LSTM: The LSTM model exhibits varying performance across different numbers of clients. For *K* = 10 clients, the best- and worst-performing clients achieved accuracies of 43.99% and 47.42% in the 1st FL round, respectively. The performance of all the clients improved with the increase in rounds. The global accuracy improved to 82.36% after the 50th FL round. Similar trends are observed for *K* = 15 and *K* = 20 clients, with accuracies ranging from 49.72% to 50.95% in the 1st FL round and global accuracies improving to approximately 82% after the 50th FL round.

**Figure 5:**
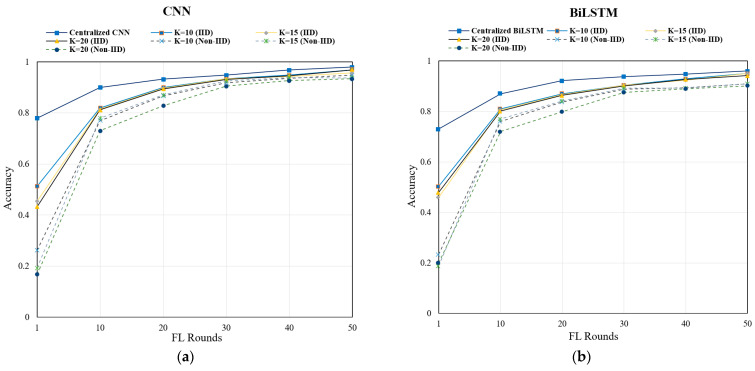

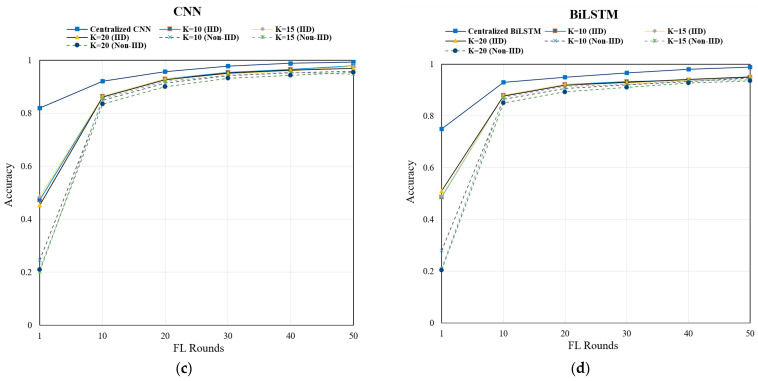
Rounds vs. accuracy of centralized vs. Federated Learning on the Edge-IIotSet dataset (**a**,**b**), and TON-IoT Dataset (**c**,**d**): (**a**) CNN Performance on Edge-IIoTSet; (**b**) BiLSTM Performance on Edge-IIoTSet; (**c**) CNN Performance on TON-IoT; and (**d**) BiLSTM Performance on TON-IoT.

We plotted the learning process vs. accuracy graph for both centralized and Federated Learning methods on the Edge-IIotSet and TON_IoT datasets, as can be seen in Figure 5. In the beginning, the gap between the centralized method (both the CNN and BiLSTM and for both IID and Non-IID data) and the Federated Learning method is quite large for both datasets. But, as we progress to the higher rounds, the gap becomes minimized, and towards the 50th round, we obtain very competitive results with the centralized methods. This signifies that our suggested FL method performs competitively in comparison to the centralized machine learning methods and has additional advantages, which we will discuss in subsequent sections.

**Figure 6:**
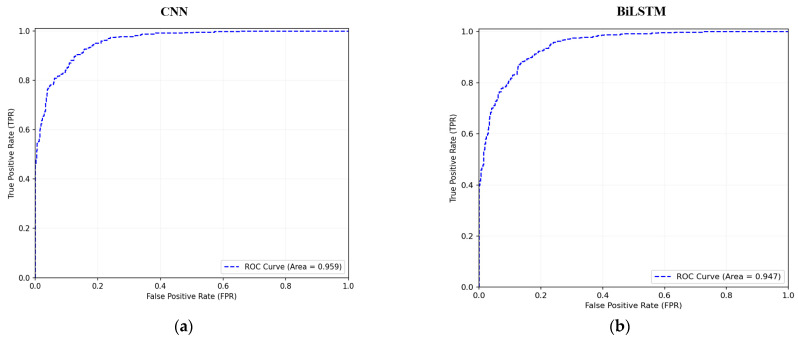

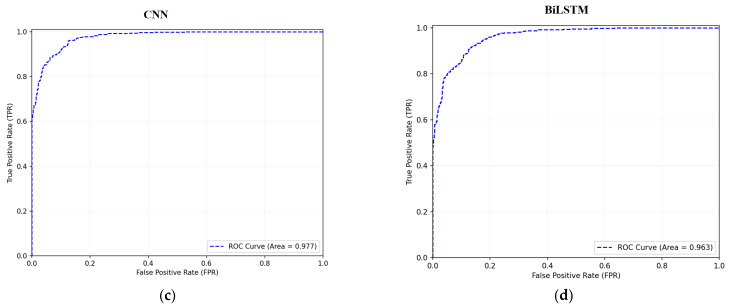
ROC curve (True Positive rate vs. False Positive rate) for the Edge-IIotSet Dataset (**a**,**b**), and TON-IoT Dataset (**c**,**d**): (**a**) CNN Performance on Edge-IIoTSet; (**b**) BiLSTM Performance on Edge-IIoTSet; (**c**) CNN Performance on TON-IoT; and (**d**) BiLSTM Performance on TON-IoT.

Figure 6 illustrates the Receiver Operating Characteristic (ROC) curves, comparing the True Positive rate against the False Positive rate for both CNN and BiLSTM models on the Edge-IIoTset and TON-IoT datasets. Figure 6a shows the CNN performance on Edge-IIoTset, with an AUC of 0.959, while Figure 6b depicts the BiLSTM performance on Edge-IIoTset, with an AUC of 0.947. Figure 6c presents the CNN performance on TON-IoT, with an AUC of 0.977, and Figure 6d shows the BiLSTM performance on TON-IoT, with an AUC of 0.963. The high AUC values, ranging from 0.947 to 0.977, indicate that both machine learning models exhibit strong performance using the proposed Federated Learning method across different datasets.

### 4.5. Deployment of Smart Contracts for the FL Distributor

In this section, we show how the smart contracts that we developed were successfully deployed, ensuring that only authorized clients could take part in the entire intrusion detection process and manage the upload and download of local and global learnings and models. We used the Remix IDE platform, as previously mentioned, to program our smart contracts in the Solidity programming language and to execute them online. We also utilized the Hardhat provider, which works as a deployment environment for the executed smart contract.

**storeRecord(uint256 Id, string memory clientId, string memory hash):**

The storeRecord function stores a new record in the contract’s storage. It takes three parameters: Id, the unique identifier of the record; clientId, the identifier associated with the client or user; and hash, the hash value representing the record’s content or data. The function first checks if the provided Id is already used by verifying the length of the clientId associated with the Id. If the Id is not already used, a new record struct is created with the provided data and stored in the record mapping with the Id as the key. Additionally, an event RecordStored is emitted to log the new record along with its details, including Id, clientId, hash, and timestamp.

**getRecord(uint256 Id) external view returns (string memory, string memory, uint256):**

The getRecord function retrieves the details of a specific record based on its unique Id. It fetches the record struct associated with the Id from the record mapping. If the record exists, meaning the length of the clientId associated with the Id is greater than zero, it returns the clientId, hash, and timestamp of the record. If the record does not exist, the function reverts with an error message indicating that the record was not found.

**Figure 7:**
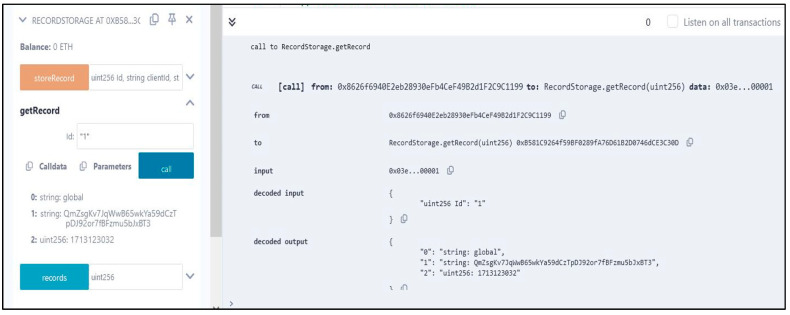
Successful deployment of smart contracts and functions.

Figure 7 shows the successful deployment and retrieval of our executed getRecord function. The ID, hash, and timestamp for that specific transaction within the Ethereum blockchain are shown.

### 4.6. Smart Contract Analysis

This subsection discusses the cost analysis of the developed smart contract functions. Gas is a currency that is utilized on the Ethereum network to complete transactions. The smart contract function’s inputs, outputs, size, and complexity all affect how much gas it uses. The execution cost is the expense related to completing various activities in the smart contract, whereas the transaction cost includes the deployment of the contract and any data moved to the blockchain network. The aim of all smart contract designs and implementations is to maximize functionality while minimizing costs. The execution and transaction costs of our suggested and implemented smart contract functions are shown in Table 4. As can be seen from the table, the costs are very low, which showcases the efficiency of our developed contracts.

**Figure 8:**
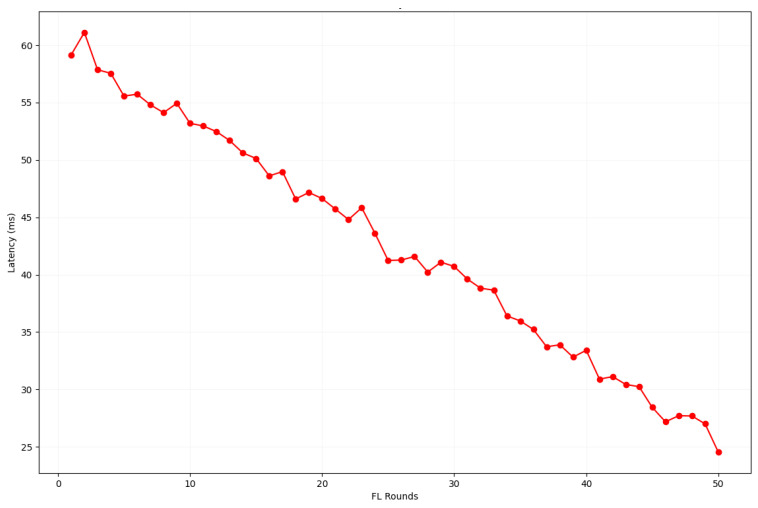
Decrease in latency with the progression of FL rounds.

In Figure 8, the decreasing latency over FL rounds reflects the system’s improved performance and efficiency in handling transactions. The trend suggests that the system can scale and handle increased workloads more efficiently over time. The consistent decrease in latency demonstrates the robustness of the system in maintaining lower latency despite the increased number of FL rounds.

### 4.7. Security Analysis of Smart Contracts

As mentioned previously, Remix IDE, which offers some code debugging and run-time error alarms, is used to develop smart contracts. They fall short of proving the reliability of smart contracts, though. It is essential to check them for reentrancy issues, looping, and other pervasive flaws that make them especially vulnerable to malevolent attackers. We investigated the embedded Ethereum smart contracts in our solution using specialized tools to check for any code vulnerabilities.

**Figure 9:**
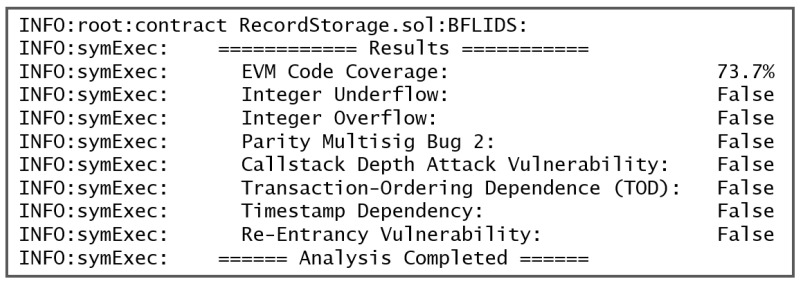
Security analysis of developed smart contracts using OYENTE.

Next, we conducted a security analysis of the smart contracts using OYENTE [57] to evaluate our developed smart contracts for our suggested BFLSID. Many different issues were evaluated by the OYENTE tool, including timestamp dependency, call stack depth attack, re-entrance vulnerabilities, and unhandled exceptions like underflow and overflow. After the smart contract has been validated, OYENTE produces a summary report, as seen in Figure 9. This code is free of these errors, as illustrated in the figure.

### 4.8. Comparison to Similar Works

In Table 5, the effectiveness of our method (BFLSID) is compared to similar Federated Learning-based Intrusion Detection Systems (FL-based IDSs). The comparison considers various aspects, including the year of deployment, whether blockchain technology is used, the dataset employed, the type of machine learning classifiers, the number of clients, accuracy, and F1-Score.

Our proposed solution stands out as the only one utilizing blockchain technology, enhancing security and privacy through smart contracts and decentralized storage systems. As shown in the table, our method achieves superior accuracy and F1-Scores compared to other methods, demonstrating its efficacy. Specifically, using the Edge_IIoTset dataset, our solution achieved an accuracy of 97.43% with CNNs and 96.02% with BiLSTM, with corresponding F1-Scores of 0.97 and 0.96. Similarly, for the TON_IoT dataset, our method reached an accuracy of 98.21% with CNNs and 97.42% with BiLSTM, with F1-Scores of 0.98 and 0.97, respectively. This performance surpasses other non-blockchain-enabled solutions listed in the table.

### 4.9. Discussion

This subsection explores the security features of the proposed blockchain-based IoT network solution and the advantages of using Federated Learning (FL) in intrusion detection for healthcare or IoMT systems.

The blockchain-based solution enhances privacy by restricting access to system phases to clients authorized through smart contracts, ensuring only selected reliable users can access stored information. Scalability is achieved through the dynamic expansion or contraction of the blockchain network based on user demand. Efficiency is improved by reducing time-consuming reporting processes through transparent architecture, leading to better synchronization and overall system quality. Comprehensive authentication and access control techniques allow participants to share information securely without exposing critical security credentials. Additionally, cryptographic hashing secures transactions, making cyberattacks like DDoS and MITM more challenging.

Federated Learning offers significant advantages for Intrusion Detection Systems. It preserves privacy by training models on decentralized data sources without sharing raw data, crucial in maintaining patient confidentiality in healthcare environments. FL supports decentralized decision-making by distributing model training across multiple edge devices, reducing reliance on a single control point. This approach also minimizes bandwidth usage as IoMT devices transmit data more securely and efficiently. FL facilitates continuous learning and adaptation to evolving threats, ensuring IDS systems remain effective over time. Moreover, FL reduces latency by enabling real-time anomaly detection through on-client predictions and allows devices to independently predict and detect intrusions even when disconnected from the network.

Integrating blockchain technology and Federated Learning enhances the security, efficiency, and robustness of Intrusion Detection Systems in IoT and healthcare environments, addressing key challenges such as privacy, scalability, and cyberattack vulnerabilities. This paper offers practical real-world applications for blockchain and FL adaptation for IoMT network intrusion detection. Our research can speed up the promotion of an ML-based product to FL with blockchain support in a production setting. Sharing resources and promoting end-device data security is also made possible by decentralized training and storage, which may lead to more effective and environmentally friendly ML-based devices.

## 5. Conclusions

In this research, we proposed a Federated Learning-driven Intrusion Detection System (BFLIDS) enhanced by blockchain technology to improve the security and privacy of IoMT networks. Our experiments using modified and improved CNN and BiLSTM models on the Edge-IIoTset and TON_IoT datasets demonstrated that our FL method achieves competitive results in intrusion detection. Our system keeps client-specific data local, sending only model updates to a decentralized storage system supported by blockchain. Smart contracts ensure authenticated and monitored transactions, while the use of IPFSs and MongoDB enhances storage security and performance.

The BFLIDS offers a range of practical applications, particularly in enhancing security across various domains. In healthcare, it can be deployed in medical facilities to secure IoMT devices, ensuring the privacy and integrity of sensitive patient data. The decentralized approach of the BFLIDS is highly scalable, making it suitable for large-scale IoT deployments in sectors such as smart homes and the industrial IoT. Additionally, by integrating blockchain and Federated Learning, our system is capable of adapting to evolving security threats, providing real-time intrusion detection and response. This adaptability ensures that the BFLIDS remains effective in protecting against unauthorized access and data breaches in a dynamic threat landscape.

Future research will focus on addressing the potential for malicious IoMT and edge nodes on the network to strengthen our proposed strategy. Incorporating advanced ML methods such as GNNs into our FL framework could potentially enhance the model’s ability to detect complex intrusion patterns. Solving FL constraints, such as clients with sparse relevant data, devices quitting mid-operation, and slow upload and model update times, can significantly improve global model accuracy. We plan to use real-time data from device-specific datasets to categorize all known and unidentified IoMT device vulnerabilities, which can be assessed using an IoMT testbed to further enhance the proposed method.

## Figures and Tables

**Table 1:** Comparison table highlighting strengths, weaknesses, and applications.

Study	Year	Strengths	Weaknesses	Applications
Zhao et al. [21]	2020	Addresses the issue of non-IID. High accuracy (99.21%), uses LSTM and RNN, and robust to long data sequences.	High computational requirements can strain devices and reduce their operational efficiency. The latency introduced by blockchain transactions may hinder the real-time processing capabilities of the IDS.	Intelligent intrusion detection in IoT networks.
Driss et al. [22]	2022	High performance in VSNs, uses GRUs, and maintains privacy. Capability to detect complex cyberattack patterns, improving the robustness of the detection system.	Specific to vehicular sensor networks and the dataset. The implementation of Federated Learning and GRU-based models can be resource-intensive.	Intrusion detection in vehicular sensor networks.
Khan et al. [23]	2020	Stackelberg game-based method and customized ML for device capabilities.	Can be complex to implement in diverse environments.	FL communication among edge servers in large IoT networks.
Nguyen et al. [24]	2019	Self-learning anomaly detection and continuous monitoring.	Potentially high False Positive rate.	Anomaly detection in IoT device communication.
Du et al. [25]	2020	Enhances security in vehicular IoT and uses MEC for large networks.	Specific to vehicular IoT devices and may not generalize well in other scenarios.	Security and performance management in vehicular IoT devices.
Mothukuri et al. [26]	2022	Maintains data privacy, uses GRUs, and is better than traditional methods.	Did not evaluate false alarm rate and few commonly used performance metrics.	Anomaly detection in IoT devices.
Wu et al. [27]	2022	Addresses fraud detection, providing a secure framework for identifying fraudulent activities in IoMT healthcare systems.	IoMT devices often have limited computational power and storage, which can hinder the performance and scalability of the proposed system.	Healthcare data management and fraud detection.
Deng et al. [28]	2021	The use of attention weights and graph-based structures provides interpretability, allowing users to understand the relationships between sensors and the root causes of detected anomalies.	The performance of the model heavily relies on the quality and completeness of the input data, which can be a limitation in environments with noisy or missing data.	Industrial IoT (IIoT) and Transportation Systems.
Xiao et al. [29]	2022	Leverages Graph Attention Networks (GATs) to capture the complex relationships between traffic data, enhancing the detection of subtle anomalies. Uses an attention mechanism within the model to provide insights into which parts of the data contribute most to anomaly detection.	Integrating GNNs into existing in-vehicle networks can be complex and resource-intensive, requiring significant computational power and expertise.	Anomaly detection in automotive security and fleet management.
Kong et al. [30]	2024	The FedCAD model demonstrates superior performance in detecting anomalies, outperforming baseline methods on benchmark datasets. The use of contrastive self-supervised learning improves the quality of data representations, leading to more accurate anomaly detection.	The current implementation is restricted to homogeneous graphs, limiting its applicability to more complex heterogeneous graph structures.	A robust framework that can handle the complexities of graph-structured data.
Poursafaei et al. [31], [32]	2021, 2022	SigTran represents nodes based on structural and transactional characteristics, effectively differentiating nodes involved in illicit activities. TGBASE does not require any parameter tuning, simplifying its implementation and use.	The integration of graph-based methods and signature vectors may introduce complexity in implementation and require substantial computational resources. TGBASE relies on a predefined set of features, which may not capture all nuances of complex temporal graphs, especially in more intricate or diverse applications.	Detecting illicit activities in blockchain transactions.
Alexopoulos et al. [33]	2017	Combines CIDSs with blockchain and enhances trustworthiness.	Managing trust among a large number of collaborating parties can be complex and resource-intensive.	Collaborative Intrusion Detection Systems (CIDSs).
Rashid et al. [34]	2021	Decentralized, secure data traceability, and combats deepfakes.	Focuses on multimedia content and does not cover detailed IoT scenarios.	Data traceability and protection against deepfakes.
Zaabar et al. [35]	2022	Uses Hyperledger Fabric, replaces the central server in FL, and secure learning.	Demonstrates implementation complexity and is specific to IoMT environments.	Intrusion detection in IoMT environments.
Casado-Vara et al. [36]	2018	Decentralized data management and uses edge computing.	High implementation complexity and uses game theory-based algorithm.	Data quality improvement and false data detection.
Alkadi et al. [37]	2020	Uses BiLSTM and smart contracts for confidentiality, outperforming existing models.	While the framework aims to minimize energy consumption and delay, it may still face challenges in highly resource-constrained environments where IoMT devices have extremely limited computational and storage capacities.	Decentralized IDSs in IoT and cloud networks.
Kumar et al. [38]	2022	Uses fog computing for DDoS detection, Random Forest, and XGBoost.	Can cause high latency in data transmission and processing in IoT networks, which can delay the detection and mitigation of threats.	DDoS detection in blockchain-enabled IoT networks.
Sindhusaranya et al. [39]	2023	Privacy-preserving, uses FL-BEPP, and addresses both soft and hard constraints.	The overhead associated with blockchain transactions and Federated Learning model updates can lead to increased latency and reduced performance in large-scale IoMT deployments.	Fraud prevention and security in the IoMT.
Golomb et al. [40]	2018	Collaborative anomaly detection utilizing blockchain.	Designed for limited resources, it may increase computation and communication overhead.	Anomaly detection in IoT networks.
Lakhan et al. [41]	2022	Considers task scheduling, energy consumption (e.g., soft constraints), and hard constraints (e.g., deadlines) when they are being executed on distributed fog and cloud nodes.	Does not address dynamic and unknown run-time attacks, which are more challenging for IoMT systems.	Fraud and privacy preservation in distributed fog and cloud nodes.
Dey et al. [42]	2018	Uses intelligent software agents and is game theory-based	Has high implementation complexity and focuses on transaction evaluation.	Identifying and evaluating malicious transactions.
Lian et al. [43]	2022	Two-stage FL approach, blockchain-based data sharing, and improves accuracy.	Data sharing adds security overheads, which could impact the overall system performance.	Collaborative model training in IoMT devices.
Eskandari et al. [44]	2023	Addresses poisoning attacks and uses a reputation-based consensus mechanism.	Has high computational requirements and is specific to poisoning attacks.	Poisoning attack prevention in FL settings.
Faheem et al. [45,46,47]	2024	These studies provide solutions combining Federated Learning and Blockchain to enhance security and privacy in IoT networks. It offers decentralized storage solutions and smart contracts for efficient data management. They also suggested blockchain for secure data traceability and privacy in multimedia content, addressing issues like deepfakes and cyberattacks.	Implementation complexity can be high, and there can be challenges in achieving scalability and managing computational overhead. The 2nd work focuses primarily on multimedia content and may not cover a broader range of IoT scenarios.	IoT networks, particularly in scenarios requiring enhanced security and privacy, such as smart grids and industrial IoT systems.

**Table 2:** Centralized models’ evaluation in intrusion detection.

Dataset	Class	Precision		Recall		F_1_-Score	
		CNN	BiLSTM	CNN	BiLSTM	CNN	BiLSTM
**Edge-IIoTset**	Normal/Benign	0.98	**0.96**	**0.99**	0.95	0.98	0.95
	Backdoor Attack	0.97	0.95	0.98	0.95	0.97	0.95
	DDoS_HTTP Attack	0.93	0.91	0.93	0.9	0.93	0.9
	DDoS_ICMP Attack	0.98	0.95	0.97	0.95	0.97	0.95
	DDoS_TCP Attack	0.84	0.8	0.85	0.81	0.84	0.8
	DDoS_UDP Attack	0.7	0.7	0.7	0.68	0.7	0.69
	Fingerprinting Attack	0.79	0.78	0.8	0.77	0.79	0.77
	MITM Attack	0.95	0.91	0.95	0.9	0.95	0.9
	Password Attack	0.84	0.85	0.84	0.85	0.84	0.85
	Port_Scanning Attack	0.99	0.96	0.99	0.96	**0.99**	**0.96**
	Ransomware Attack	0.89	0.88	0.89	0.87	0.89	0.87
	SQL_injection Attack	0.72	0.71	0.73	0.66	0.72	0.68
	Uploading Attack	0.79	0.76	0.78	0.77	0.78	0.76
	Vulnerability_scanner	**0.99**	0.95	0.98	**0.96**	0.98	0.95
	XSS Attack	0.84	0.82	0.83	0.82	0.83	0.82
**TON_IoT**	Normal	0.98	0.97	0.98	0.95	0.98	0.96
	DDoS	0.99	0.97	0.98	0.96	0.99	0.96
	Injection	0.96	0.94	0.97	0.94	0.97	0.94
	Password	0.95	0.94	0.96	0.93	0.96	0.93
	Backdoor	0.98	0.97	0.97	0.95	0.98	0.96
	Ransomware	0.99	0.97	0.98	0.96	0.99	0.97
	XSS	0.99	0.98	0.98	0.96	0.99	0.98
	Scanning	0.99	0.99	0.99	0.96	0.99	0.97

**Table 3:** Federated Learning model’s evaluation for intrusion detection.

Dataset	Classifier	Clients	1st FL Round						50th FL Round					
			IID			Non-IID			IID			Non-IID		
			Best	Worst	Global	Best	Worst	Global	Best	Worst	Global	Best	Worst	Global
**Edge-IIoTset**	**CNN**	*K* = 10	0.53	0.38	0.51	0.28	0.13	0.26	0.98	0.96	0.97	0.96	0.94	0.95
		*K* = 15	0.46	0.35	0.45	0.20	0.09	0.19	0.98	0.95	0.96	0.95	0.92	0.94
		*K* = 20	0.46	0.32	0.43	0.19	0.06	0.17	0.99	0.95	0.97	0.95	0.91	0.93
	**BiLSTM**	*K* = 10	0.42	0.44	0.50	0.25	0.17	0.23	0.96	0.94	0.96	0.92	0.90	0.91
		*K* = 15	0.48	0.40	0.46	0.21	0.13	0.19	0.96	0.94	0.95	0.92	0.90	0.91
		*K* = 20	0.49	0.42	0.48	0.21	0.14	0.20	0.97	0.94	0.94	0.91	0.89	0.90
**TON_IoT**	**CNN**	*K* = 10	0.57	0.41	0.47	0.32	0.16	0.25	0.99	0.95	0.98	0.97	0.94	0.96
		*K* = 15	0.48	0.38	0.48	0.30	0.14	0.20	0.99	0.94	0.98	0.96	0.95	0.95
		*K* = 20	0.48	0.34	0.45	0.21	0.10	0.21	0.99	0.96	0.97	0.96	0.94	0.95
	**BiLSTM**	*K* = 10	0.44	0.47	0.49	0.31	0.07	0.28	0.93	0.90	0.93	0.95	0.91	0.95
		*K* = 15	0.50	0.43	0.49	0.26	0.20	0.21	0.95	0.90	0.93	0.95	0.92	0.93
		*K* = 20	0.51	0.45	0.51	0.27	0.15	0.20	0.94	0.89	0.93	0.93	0.90	0.92

**Table 4:** Cost analysis of executed smart contract functions.

Functions	Transaction Cost (Gas)	Execution Cost (Gas)
*StoreRecord*	31,845	25,158
*GetRecord*	43,738	29,184

**Table 5:** Comparison of the proposed method to similar works.

IoT IDS	Year	Blockchain-Enabled?	Dataset	Classifier	No. of Clients	Accuracy (%)	F1-Score
Li et al. [58]	2021	No	Gas Pipeline	CNN-GRU	*K* = [3, 5, 7]	96.20	0.95
Zhao et al. [21]	2020	No	SEA	RNN-LSTM	*K* = 4	97.21	0.96
Nguyen et al. [24]	2019	No	Private Dataset	RNN-GRU	*K* = [5, 9, 15]	96.51	0.96
Huong et al. [59]	2021	No	Bot-IoT	LocKedge	*K* = 4	96.70	0.95
Khan et al. [60]	2024	No	ToN_IoT	RL (Q-Learning)	*K*= 3	96.40	0.95
**Our Solution**	**2024**	**Yes**	**Edge_IIoTset**	**CNN, BiLSTM**	***K* = [10, 15, 20]**	**97.43 (CNN)** **96.02(BiLSTM)**	**0.97 (CNN)** **0.96(BiLSTM)**
			**TON_IoT**	**CNN, BiLSTM**	***K* = [10, 15, 20]**	**98.21(CNN)** **97.42(BiLSTM)**	**0.98 (CNN)** **0.97(BiLSTM)**

## Data Availability

The original data presented in this study are openly available in [kaggle.com and paperswithcode.com] at [URL: https://www.kaggle.com/datasets/mohamedamineferrag/edgeiiotset-cyber-security-dataset-of-iot-iiot/data and URL: https://paperswithcode.com/dataset/ton-iot]. URL (accessed on 12 April 2024).
